# A Morphometric Study of the Adult Distal Femur in the South Indian Population

**DOI:** 10.7759/cureus.69289

**Published:** 2024-09-12

**Authors:** Janani S V, Kalpana Ramachandran

**Affiliations:** 1 Anatomy, Sri Ramachandra Institute of Higher Education and Research, Chennai, IND

**Keywords:** femoral condyles, joint replacement surgery, morphometry, osteoarthritis, prosthesis design, surgical outcomes

## Abstract

Introduction

The most vital joint for locomotion is the knee joint, which is a condylar, modified hinge joint. Osteoarthritis is a degenerative disease commonly affecting the knee joint, which can be successfully treated by joint replacement surgeries wherein the condyles of the affected knee joints are replaced based on the measurement of the condyles for which the accurate morphometric values of the tibia and femur play an important role thereby decreasing the complications post-surgery and improving the mobility and quality of life of patients.

Aim

The present study aims to evaluate the morphometric data of femoral condyles and compare the morphometric data of the left and right femurs using the direct method.

Materials and methods

One hundred femoral condyles of unidentified sex were used for the measurement in the study. Medial condylar anteroposterior distance (MCAPD), lateral condylar anteroposterior distance (LCAP), bicondylar width (BCW), medial condylar transverse distance (MCTD), lateral condylar transverse distance (LCTD), and intercondylar notch width (ICNW) were measured by Vernier Calliper and the values between the left and right femurs were compared.

Results

The mean MCAPD is 56.88 mm, the mean LCAP is 57.72 mm, the mean BCW is 72.22 mm, the mean MCTD is 22.88 mm, the mean LCTD is 22.99 mm, and the mean ICNW is 21.58 mm, respectively.

Conclusion

The morphometric data obtained by direct measurement using Vernier Callipers is more accurate than indirect measurements which in turn aid biomedical engineers in designing more accurate and apt prostheses for knee replacement surgeries which in turn decrease the post-surgical complications and result in better outcomes due to surgery.

## Introduction

The femur holds the distinction of being both the longest and strongest bone in the human body. It helps in the transmission of weight from the ileum to the upper end of the tibia through an unstable bony arrangement at the knee joint. The femur's length correlates with the striding gait, its strength with weight, and muscular forces [1]. Its shaft is mostly cylindrical and bows forward while its proximal end features a rounded, articular head projecting medially from a short neck, which is itself a medial extension of the proximal shaft. The lower end of the femur is broader and more robust, featuring a widely expanded double condyle bearing partly articular surface for transmission of weight to the tibia [2]. The condyles merge anteriorly, extending into the shaft, while posteriorly they are divided by a deep intercondylar fossa and extend beyond the plane of the popliteal surface. The articular surface is a broad area, like an inverted U, for the patella and the tibia.

Osteoarthritis (OA) is a degenerative joint disease characterized by the breakdown of cartilage in the joints, leading to pain, stiffness, and reduced mobility. It is the most common form of arthritis, and one of the joints commonly affected is the knee. As the cartilage wears away, bones may rub against each other, causing pain and inflammation. OA of the knee often progresses slowly, and its prevalence increases with age [3].

Total knee arthroplasty (TKA), commonly known as knee replacement surgery, is a surgical procedure designed to alleviate the pain and disability associated with severe knee OA [4]. During TKA, the damaged or worn-out parts of the knee joint are replaced with artificial components, including a femoral component, a tibial component made of metal, and a plastic spacer. The goal is to restore function, reduce pain, and improve the overall quality of life for individuals suffering from advanced OA [4].

Hence this knee replacement surgery requires proper implants of adequate size for joint mobility, eliminating pain, improving the quality of life, and preventing complications post-surgery [5]. Indirect morphometric studies done through X-ray, MRI, and CT scan are inaccurate in most instances and this may further increase the probability of occurrence of complications. Hence direct morphometric studies are more effective for better surgical outcomes [6].

The present study is based on the difference between the ethnic groups and the side of the femur in the South Indian population. The data obtained via this study will help in the determination of the appropriate size of the implant and thereby help in preventing complications that may arise due to TKA.

## Materials and methods

**Figure 1:**
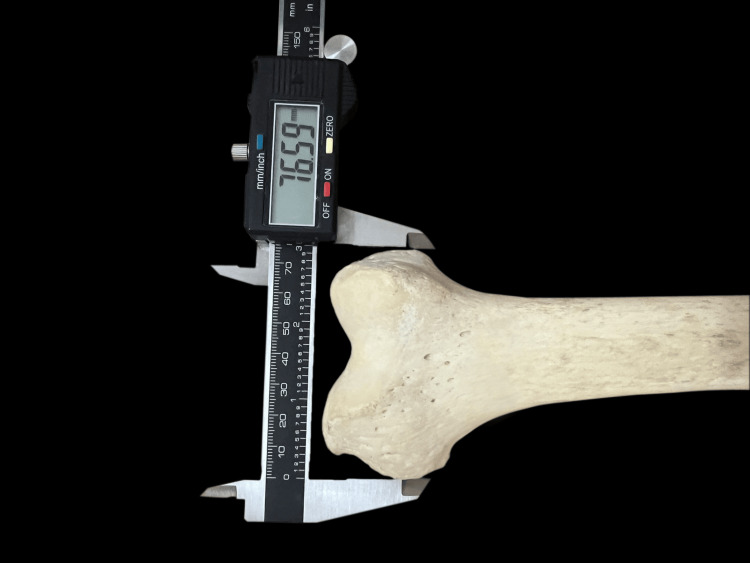
Measurement of BCW using Sliding Digital Calliper BCW, bicondylar width

The present study has been conducted in the Department of Anatomy, Sri Ramachandra Institute of Higher Education and Research. The study was done after getting approval from the Institutional Ethics Committee. The study included measurements on the femur of unpaired dry adult bones. The dry bones (100 femurs: 50 right and 50 left) were obtained from the Department of Anatomy, Sri Ramachandra Medical College, Chennai. Bones having deformity, fractures, arthritic changes, unfused epiphyses, and macerated condyles were not included in the study. The measurements (bones) were taken using the Sliding Digital Caliper with a resolution of 0.01 mm (Figure 1).

**Figure 2:**
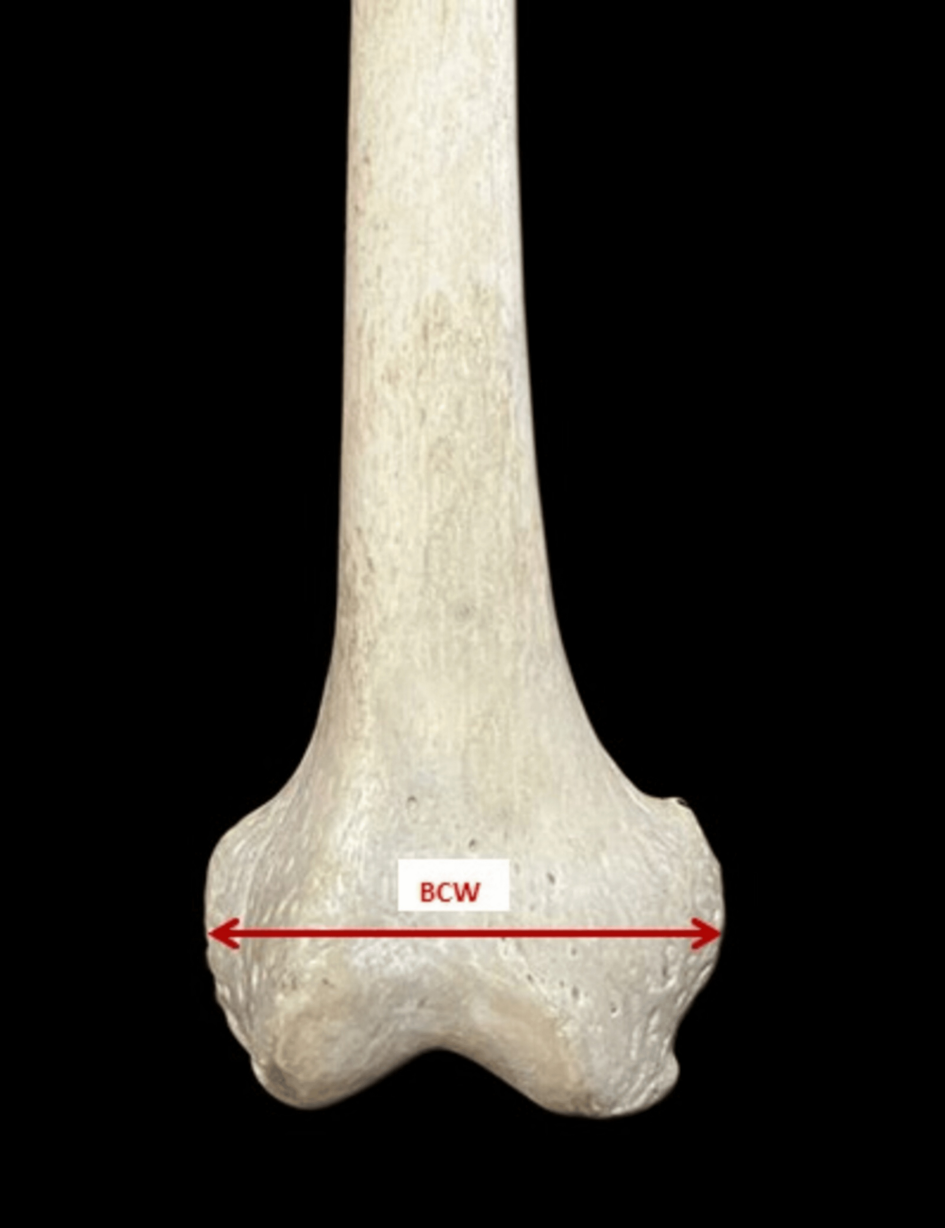
Measurement of BCW BCW, bicondylar width

**Figure 3:**
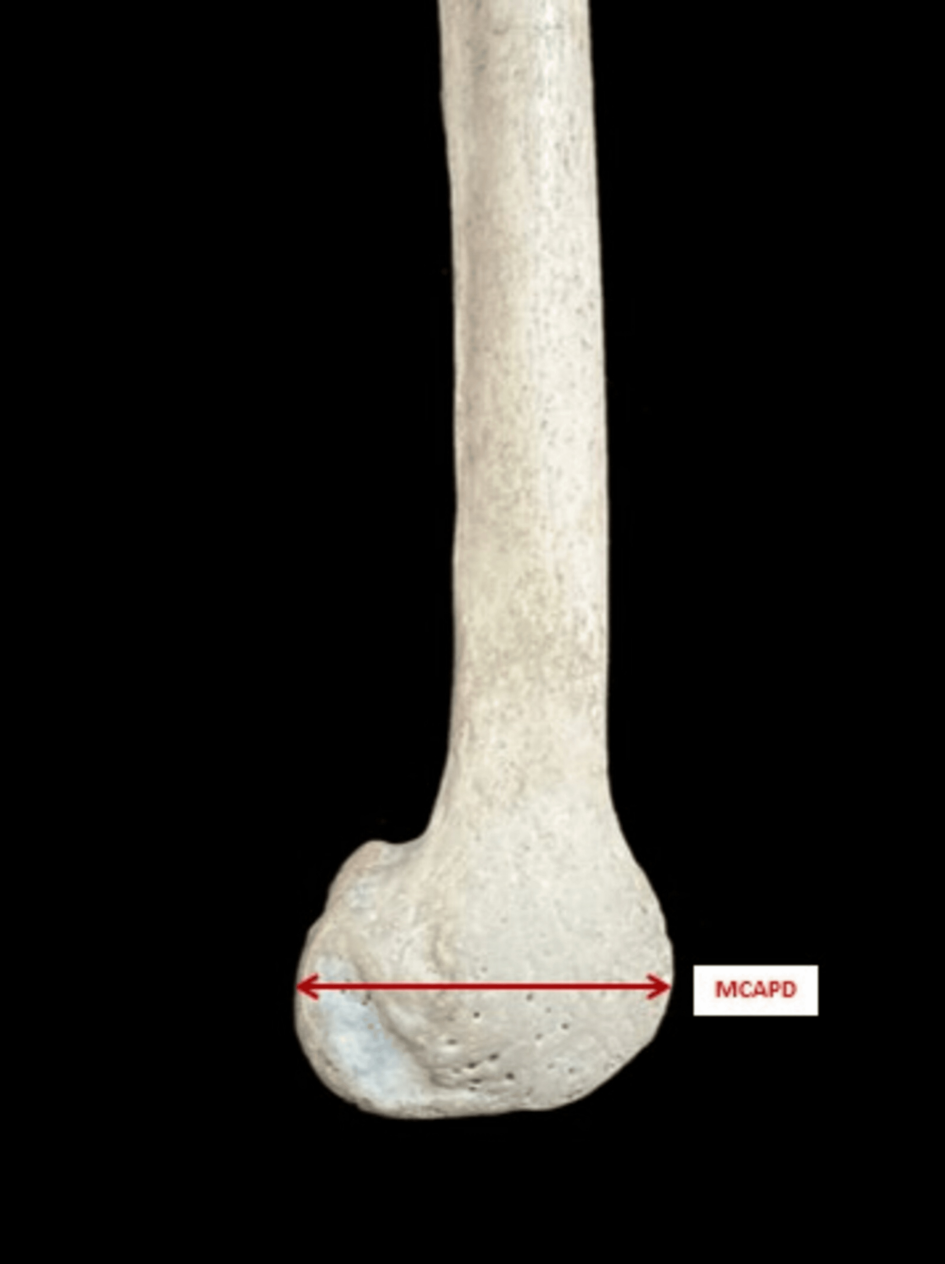
Measurements of MCAPD MCAPD, medial condylar anteroposterior distance

**Figure 4:**
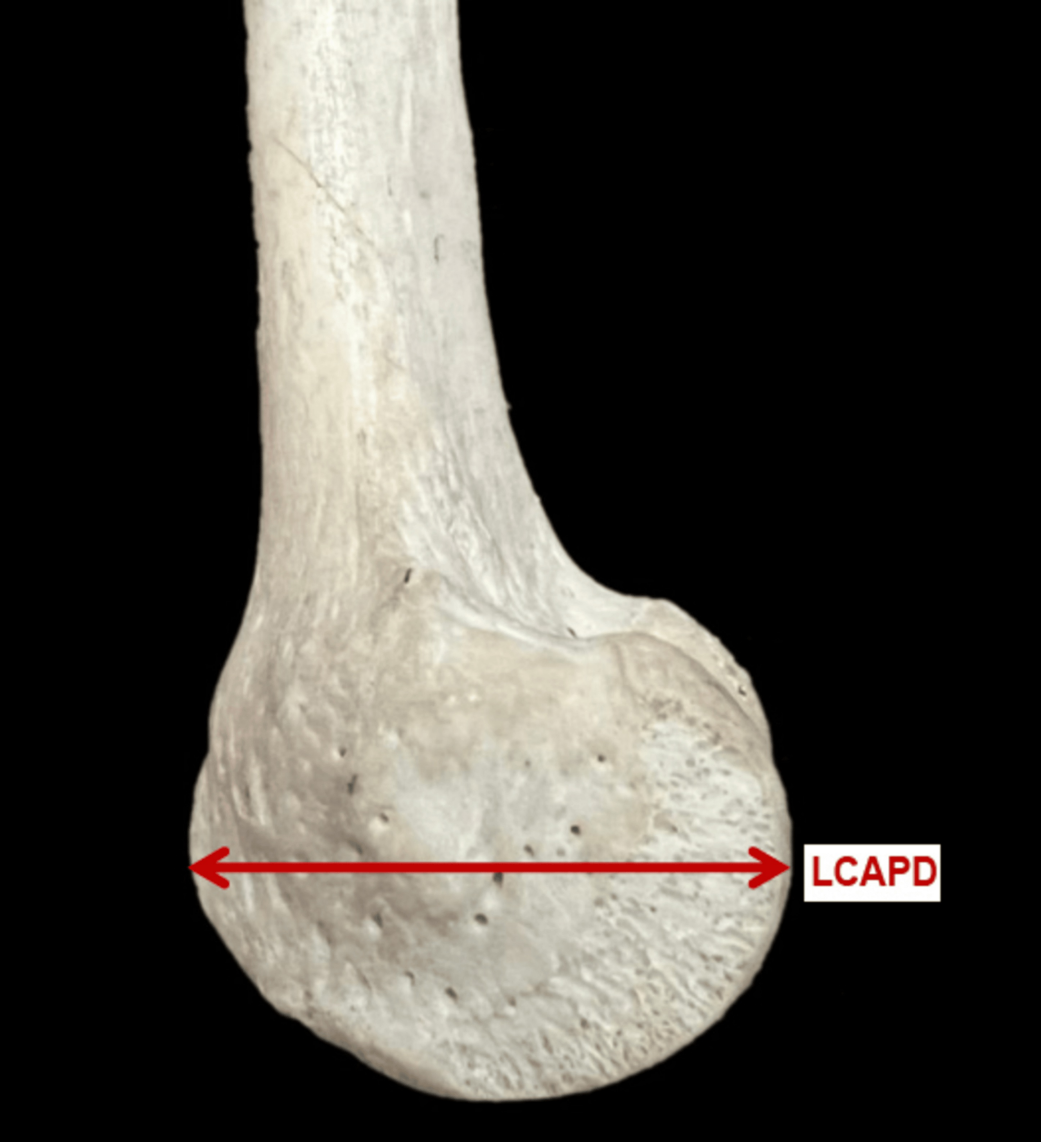
Measurement of LCAP LCAP, lateral condylar anteroposterior distance

**Figure 5:**
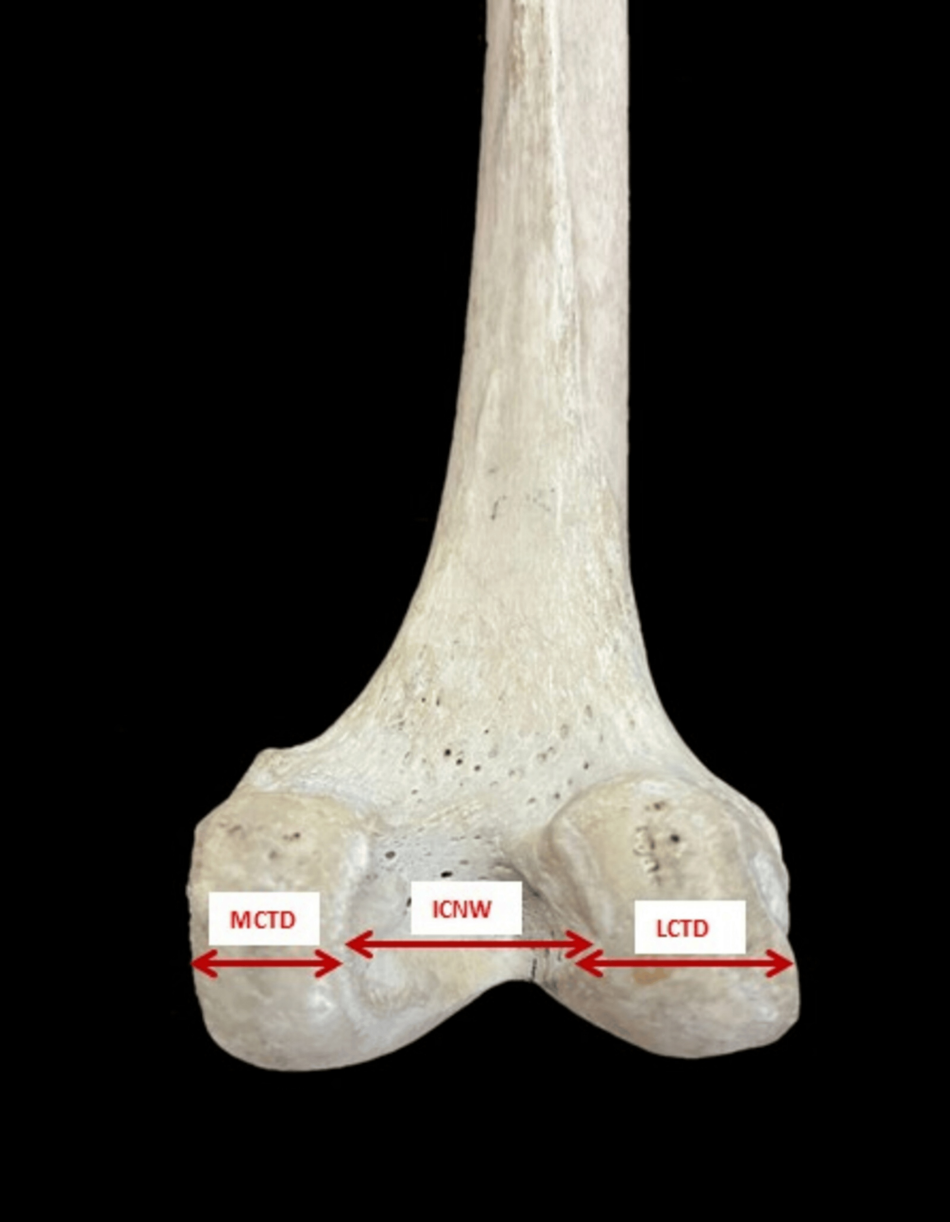
Measurement of ICNW and medial and lateral transverse diameter ICNW, intercondylar notch width

The parameters measured are as follows: 1. The bicondylar width (BCW) - the maximum distance between the medial and lateral epicondyle in the lateral plane (Figure 2). 2. The lateral condylar anteroposterior distance (LCAPD) - the maximum distance between the anterior and posterior surface of the lateral condyle (Figure 3). 3. The medial condylar anteroposterior distance (MCAPD) - the maximum distance between the anterior and posterior surface of the medial condyle (Figure 4). 4. The intercondylar notch width (ICNW) - the maximum distance between the medial and lateral surface of the intercondylar notch posteriorly (Figure 5). 5. The medial condylar transverse distance (MCTD) - the maximum distance between the medial and lateral surface of the medial condyle (Figure 5). 6. The lateral condylar transverse distance (LCTD) - the maximum distance between the medial and lateral surface of the lateral condyle (Figure 5).

## Results

Bicondylar width of femur

It is observed that out of the 50 femurs studied, the BCW of the left femur ranges from 60.0 to 89.9 mm, with the highest proportion of 36% within 70.0-74.9 mm (Table 1). The mean BCW for the left femur is recorded as 72.82 mm, with a standard deviation of 3.89 mm. Conversely, the BCW of the right femur spans the same range of 60.0 to 89.9 mm, with the highest proportion of 32% within 65.0 to 69.9 mm (Table 1). The mean BCW for the right femur is slightly lower at 71.62 mm, with a higher standard deviation of 5.67 mm, indicating greater variability in measurements compared to the left femur (Table 2).

**Table 1:** BCW of left side and right side BCW, bicondylar width

Left side (N=50)			Right side (N=50)	
BCW	Frequency in numbers	Percentage in total	Frequency in numbers	Percentage in total
60.0-64.9 mm	1	2	3	6
65.0-69.9 mm	11	22	16	32
70.0-74.9 mm	18	36	13	26
75.0-79.9 mm	17	34	13	26
80.0-84.9 mm	2	2	4	8
85.0-89.9 mm	1	1	1	2
Total	50	100	50	100

**Table 2:** Comparison of BCW of the left and right sides BCW, bicondylar width

Side	Number of femurs studied	Mean	Standard deviation
Left	50	72.82	3.89
Right	50	71.62	5.67
Total	100	72.22	4.88

Lateral condylar anteroposterior distance

The LCAP of the left femur shows a mean measurement of 58.52 mm, with a standard deviation of 3.96 mm. The majority of measurements fall within the ranges of 55.0-59.9 mm and 60.0-64.9mm, with 36% and 46% of observations, respectively (Table 3). Comparatively, the right femur exhibits a mean measurement of 56.92 mm, with a standard deviation of 2.17 mm. Measurements on the right femur predominantly cluster within the ranges of 55.0-59.9mm and 60.0-64.9mm, with 33% and 38% of observations, respectively (Table 3). This indicates a slightly larger mean LCAP for the left femur compared to the right femur, alongside greater variability in measurements for the left femur (Table 4).

**Table 3:** LCAP of the left and right femurs LCAP, lateral condylar anteroposterior distance

Left femur (n=50)			Right femur (n=50)	
LCAP distance	Frequency in numbers	Percentage in total	Frequency in numbers	Percentage in total
50.0-54.9mm	9	18	15	30
55.0-59.9mm	18	36	16	33
60.0-64.9mm	23	46	19	38
Total	50	100	50	100

**Table 4:** Comparison of LCAP the between left and right sides LCAP, lateral condylar anteroposterior distance

Side	Number of femurs studied	Mean	Standard deviation
Left	50	58.52	3.44
Right	50	56.92	4.41
Total	100	57.72	4.01

Medial condylar anteroposterior distance

The MCAP of the left femur ranges from 45.0 to 64.9 mm, with the highest proportion of 38% within 55.0 to 59.9 mm (Table 5). The mean medial anteroposterior distance for the left femur is recorded as 56.62 mm, with a standard deviation of 4.19 mm. Similarly, for the right femur, the MCAP spans the same range of 45.0 to 64.9 mm, with the highest proportion of 41% within 55.0 to 59.9 mm (Table 5). The mean medial anteroposterior distance for the right femur is slightly larger at 57.14 mm, with a standard deviation of 4.82 mm (Table 6). This comparison reveals a marginally larger mean medial anteroposterior distance for the right femur and slightly more variability in measurements for the right femur compared to the left femur.

**Table 5:** MCAPD of the left and right femurs MCAPD, medial condylar anteroposterior distance

Left femur (n=50)			Right femur (n=50)	
MCAP distance	Frequency in numbers	Percentage in total	Frequency in numbers	Percentage in total
45.0-49.9mm	2	4	3	6
50.0-54.9mm	14	28	12	24
55.0-59.9mm	19	38	21	41
60.0-64.9mm	15	30	14	28
Total	50	100	50	100

**Table 6:** Comparison of MCAPD between the left and right sides MCAPD, medial condylar anteroposterior distance

Side	Number of femurs studied	Mean	Standard deviation
Left	50	56.62	4.19
Right	50	57.14	4.82
Total	100	56.88	4.5

Lateral condylar transverse distance

The frequency distribution of the LCTD in the left femur indicates measurements ranging from 15.0 to 29.9 mm, with 58% within 20.0 to 24.9 mm (Table 7). Similarly, for the right femur, measurements span the same range, with 64% within 20.0 to 24.9 mm (Table 7). The mean lateral transverse distance for the right femur is slightly larger at 23.12 mm compared to the left femur's mean of 22.64 mm (Table 8). Additionally, the standard deviation for the left femur is higher at 3.96 mm compared to the right femur's standard deviation of 2.17 mm, indicating greater variability in measurements for the left femur.

**Table 7:** LCTD of the left and right femurs LCAP, lateral condylar anteroposterior distance

Left femur (n=50)			Right femur (n=50)	
LCTD distance	Frequency in numbers	Percentage in total	Frequency in numbers	Percentage in total
50.0-54.9mm	9	18	15	30
55.0-59.9mm	18	36	16	33
60.0-64.9mm	23	46	19	38
Total	50	100	50	100

**Table 8:** Comparison of LCTD between the left and right sides LCTD, lateral condylar transverse distance

Side	Number of femurs studied	Mean	Standard deviation
Left	50	58.52	3.44
Right	50	56.92	4.41
Total	100	57.72	4.01

Medial condylar transverse distance

The frequency distribution of the MCTD in the left femur reveals measurements spanning from 15.0 to 29.9 mm, with 58% within 20.0-24.9 mm (Table 9). The mean medial transverse distance for the left femur is recorded as 22.64 mm, with a standard deviation of 3.96 mm. Similarly, for the right femur, measurements range from 15.0 to 29.9 mm, with 72% within 20.0-24.9 mm (Table 9). The mean medial transverse distance for the right femur is slightly larger at 23.12 mm, with a smaller standard deviation of 2.17 mm (Table 10). This comparison illustrates a slightly larger mean medial transverse distance for the right femur and a smaller standard deviation, indicating less variability in measurements compared to the left femur.

**Table 9:** MCTD of the left and right femurs MCTD, medial condylar transverse distance

Left femur (n=50)			Right femur (n=50)	
MCTD	Frequency in numbers (lt)	Percentage in total	Frequency in numbers (rt)	Percentage in total
15.0-19.9 mm	5	10	2	4
20.0-24.9 mm	29	58	36	72
25.0-29.9 mm	16	32	12	24
Total	50	100	50	100

**Table 10:** Comparison of MCTD between the left and right sides MCTD, medial condylar transverse distance

Side	Number of femurs studied	Mean	Standard deviation
Left	50	56.62	4.19
Right	50	57.14	4.82
Total	100	56.88	4.5

Intercondylar notch width

The frequency distribution of the ICNW in the left femur indicates measurements ranging from 15.0 to 29.9 mm, with 74% within 20.0-24.9 mm (Table 11). Similarly, for the right femur, measurements span the same range, with 54% within 20.0-24.9 mm (Table 11). The mean ICNW for the right femur is slightly larger at 23.12 mm compared to the left femur's mean of 21.66 mm. Additionally, the standard deviation for the right femur is higher at 4.64 mm compared to the left femur's standard deviation of 2.69 mm, indicating greater variability in measurements for the right femur (Table 12).

**Table 11:** Intercondylar width of the left and right femurs

Intercondylar width (mm)	Frequency in numbers	Percentage in total	Frequency in numbers	Percentage in total
60.0-64.9mm	1	2	3	6
65.0-69.9mm	11	22	16	32
70.0-74.9mm	18	36	13	26
75.0-79.9mm	17	34	13	26
80.0-84.9mm	2	2	4	8
85.0-89.9mm	1	1	1	2
Total	50	100	50	100

**Table 12:** Comparison of intercondylar width of the left and right sides

Side	Number of femurs studied		Mean		Standard deviation	
Left		50		72.82		3.89
Right		50		71.62		5.67
Total		100		72.22		4.88

## Discussion

The femur is the longest and strongest bone in the human body. It supports the body's weight and facilitates various movements. The femur articulates with the tibia and patella to form the knee joint at its distal end. It is a complex structure essential for weight-bearing and mobility. Understanding the anatomy of the distal end of the femur plays a crucial role in diagnosing and treating various knee-related conditions and injuries [7].

The femur is exposed to a variety of traumatic, inflammatory, degenerative, and neoplastic processes due to its position, structure, and function. Therefore, a comprehensive understanding of femur anatomy, including surface contours, curvatures, and dimensions, is crucial for comprehending disease processes and effectively managing and reconstructing joint and fracture surgeries [8]. This anatomical knowledge forms the foundation for ensuring appropriate treatment and successful outcomes in surgical interventions related to the femur.

The distal end of the femur features two primary articular surfaces. The medial condyle articulates with the medial condyle of the tibia, forming the medial compartment of the knee joint. It possesses a smooth, convex surface facilitating smooth movement during knee flexion and extension. Conversely, the lateral condyle, located opposite to the medial condyle, articulates with the lateral condyle of the tibia, forming the lateral compartment of the knee joint. Similar to the medial condyle, it also exhibits a smooth, convex surface enabling proper articulation and movement. Between these condyles lies the intercondylar notch, a V-shaped depression on the distal femur serving as a passageway for crucial structures like the anterior and posterior cruciate ligaments, vital for knee joint stabilization [9]. Anteriorly, the distal femur features a smooth, grooved surface known as the patellar surface, articulating with the patella, which is embedded within the quadriceps tendon. This interaction enables the proper functioning of the patellofemoral joint during activities such as walking, running, and climbing stairs. Additionally, the medial and lateral epicondyles, located on either side of the distal femur, serve as attachment points for various ligaments and tendons, crucial for stabilizing the knee joint and transmitting forces during movement. Moreover, the medial and lateral condyles of the distal femur exhibit prominent ridges known as condylar ridges, providing additional stability to the knee joint and serving as attachment sites for the menisci, fibrocartilage structures enhancing joint congruence and load distribution during weight-bearing activities [10].

The knee is the largest and most intricate joint in the human body, and it is highly susceptible to frequent injury. Several factors contribute to this high incidence, including its complex diarthrodial structure and its position between the body's longest lever arms, the femur and the tibia, making it particularly vulnerable to sports-related injuries. The knee joint serves two opposing functions: mobility and stability, which adds to its complexity. It consists of the distal end of the femur, the proximal end of the tibia, and the patella, operating under axial compression due to gravity. This complexity increases its vulnerability to injury. The joint's stability is enhanced by the convex shape of the condyles in both planes, the distal extension of the medial condyle, and the more prominent lateral condyle, which prevents lateral displacement of the patella. The knee joint functions as a modified hinge joint, facilitating movement primarily in one direction, flexion and extension. With longer life spans, osteoporosis and wear of weight-bearing joints have become increasingly common. Advances in imaging and prosthetic technology have popularized total knee replacement surgeries. The key to successful knee arthroplasty lies in precise morphometric data and the selection of prostheses that are geometrically and size-appropriately matched. Therefore, accurate morphometric measurements are essential before choosing the implant size to ensure optimal fit and function of the prosthesis, crucial for the long-term success and functionality of knee replacements [11].

This present study estimates six parameters from the lower end of the dry femur and the data has been collected using direct Vernier Calliper measurements as the indirect methods of measurements are found to be inaccurate in most cases. The data found in this study is in accordance with the study conducted by Terzidis et al. [6]. A study conducted by Neelima et al. found slightly lower values compared to our study with an anteroposterior length of the medial condyle as 57.83 mm, the width of the medial condyle as 21.33 mm, anteroposterior length of the lateral condyle as 58 mm, width of the lateral condyle as 21.08 mm, and ICNW as 22.83 mm [7]. A similar study has been conducted on the Iranian population by Moghtadaei et al., which showed results consistent with the present study. Direct measurement has the advantage of giving accurate measurement thereby aiding in appropriate prosthetics for joint replacement surgeries with lesser incidence of complications [12].

The variations in the values can be attributed to lifestyle modifications in the South Indian population. South Indian population has a more sedentary lifestyle, which is the main contributing factor to the development of degenerative changes. Other factors include genetics, environmental changes, stature, and composition of the human body. A study conducted by Biswas et al. among the Bengali population established similar findings to our study [8]. A study conducted by Herzog et al. suggests that if there is no statistical significance then the contralateral healthy side can be used for the pre-operative measurements during knee replacement surgeries (Table 13) [13].

**Table 13:** Comparison of results between various studies BCW, bicondylar width; MCAPD, medial condylar anteroposterior distance; LCAPD, lateral condylar anteroposterior distance; MCTD, medial condylar transverse distance; LCTD, lateral condylar transverse distance; ICW, intercondylar width

Year of study and population studied	BCW		MCAPD		LCAPD		MCTD		LCTD		ICW	
	R	L	R	L	R	L	R	L	R	L	R	L
Mistri Set al., 2015, West Bengal, 127 bones (65 R, 62 L) [14]	74.43±6.10	73.96±5.99	-	-	-	-	-	-	-	-	19.12±2.5	18.65±2.8
Terzidis et al., 2012, Greek, 360 bones (180R, 180L) [6]	84.1±0.62	83.7±0.63	58.6±4.1	58.7±4.1	58.5±4.0	-	-	-	-	-	20.5±2.3	20.5±2.2
Ameet KJ et al., 2014, 97 bones (45R, 52 L) [15]	72.5±5.3	73.3±5.3	-	-	-	-	-	-	-	-	18.0±3.0	17.9±2.5
Shweta Jet al., 2017, North India, 100 bones (51R, 49 L) [10]	73.1±6.14	72.16±6.58	-	-	-	-	-	-	-	-	20.82±2.57	21.03±3.13
Biswas A et al., 2017, West Bengal, 70 bones (35R, 35L) [8]	71.71±4.50	70.71±5.25	52.97±3.77	54.74±3.85	56.20±3.36	56.05±4.29	25.48±2.05	27.28±2.29	27.80±2.91	28.03±2.56	20.86±2.52	19.45±2.57
Present study	71.62±5.67	72.82±3.89	57.14±4.82	56.62±4.19	58.52±3.44	56.92±4.41	23.12±2.17	22.64±3.96	23.12±2.34	22.86±3.12	21.5±4.64	21.66±2.69

## Conclusions

Accurate measurements of the parameters are a necessity for the designing of better-fitting prostheses in knee replacement surgeries. Direct measurement using Vernier Calipers is one of the most accurate tools for measurement of the various condylar diameters of the knee joint. The results are conclusive of appropriately accurate measurements that can help with better outcomes in knee replacement surgeries in degenerative disorders of the knee joint and play a vital role in minimizing the complications and improving the quality of life of these patients.
